# Supplementary material for: A case of T-cell acute lymphoblastic leukemia in retroviral gene therapy for ADA-SCID
Source: Nat Commun. 2024 Apr 30;15:3662. doi: 10.1038/s41467-024-47866-5 (PMC11061298; doi:10.1038/s41467-024-47866-5)
Supplement: Supplementary file 1 — Supplementary Information [file 41467_2024_47866_MOESM1_ESM.pdf]

Supplementary Figure 1

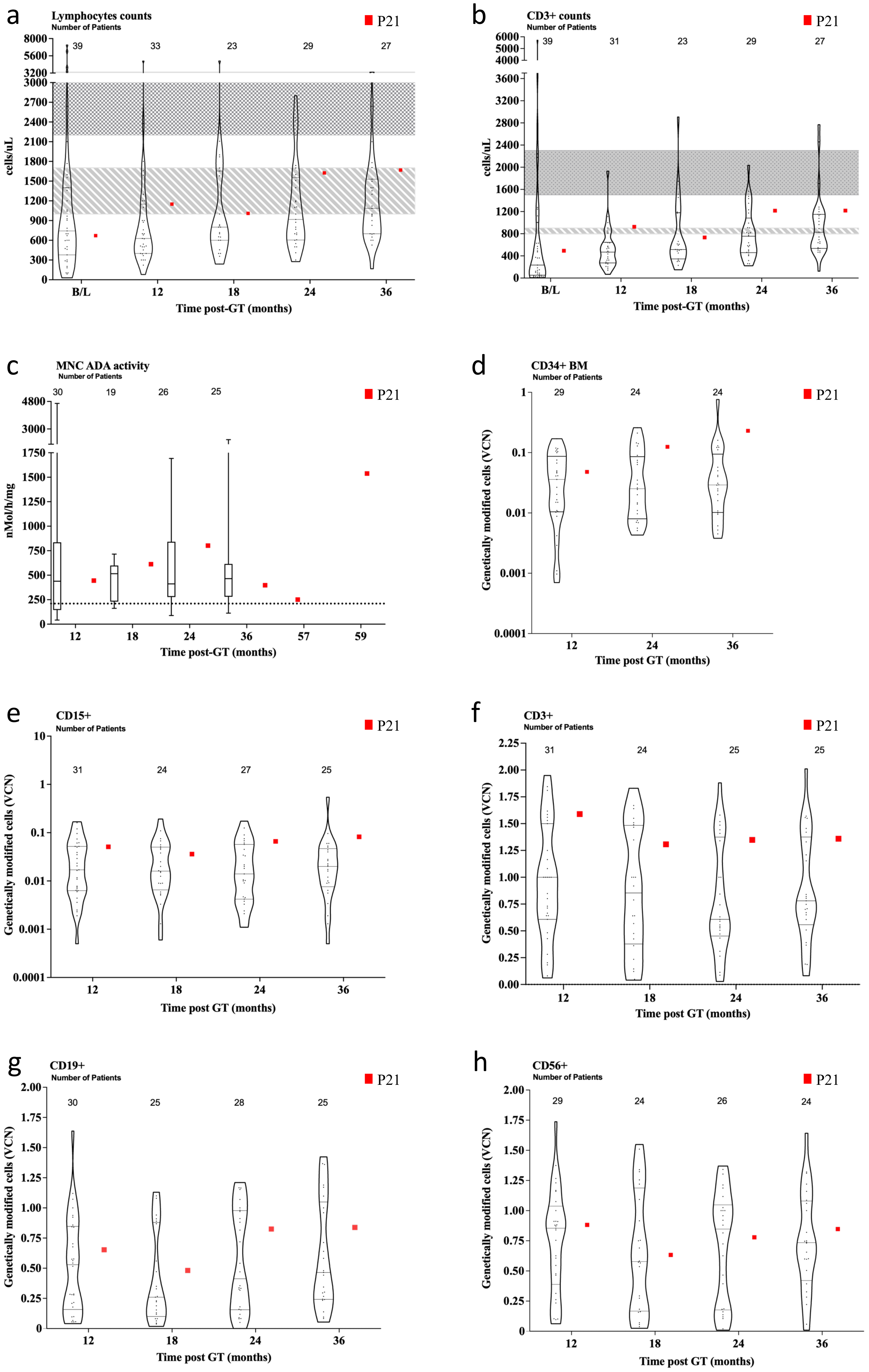

Supplementary Figure 1: Immune reconstitution, In vivo engraftment of genetically corrected cells and metabolic detoxification of P21 after gene therapy.

a-b) Immune reconstitution of P21 is compared to that of other ADA SCID patients undergoing γRV-GT. Lymphocytes (a) and CD3+ (b) are reported as cells/μL in linear scale. The shaded dark and light grey regions represent median and fifth percentile values, respectively, in normal children. The top edges correspond to levels in children ages 2 to 5 years; bottom edges correspond to levels in children ages 10 to 16 years. Values for children ages 5 to 10 typically fall within the shaded areas. B/L: baseline.

c) ADA activity levels measured on mononuclear cells (MNC) of P21, compared to those of other γRV-GT ADA SCID patients. Dashed line indicates the lower reference value of ADA activity for patients undergone successful hematopoietic stem cell transplantation.

GT; d-e) Bone marrow CD34+ cells (d) and peripheral CD15+ (e) gene-modified cells of P21 were log-transformed, plotted on a linear axis (with antilog ticks) and compared to those of other ADA SCID patients who undergone γRV-GT. f-h) Peripheral CD3+ (f), CD19+ (g) and CD56+ (h) gene-modified cells of P21, compared to other ADA SCID patients undergone γRV-GT, were reported in linear scale. From 2000 to 2012, the frequency of transduced cells and vector copy number were determined on genomic DNA by quantitative PCR analysis for NeoR vector sequences, normalized for DNA content. Since the end of 2012, the method changed to RT-PCR for the evaluation of VCN/genome. VCN: vector copy number; B/L: baseline.

Supplementary Figure 2

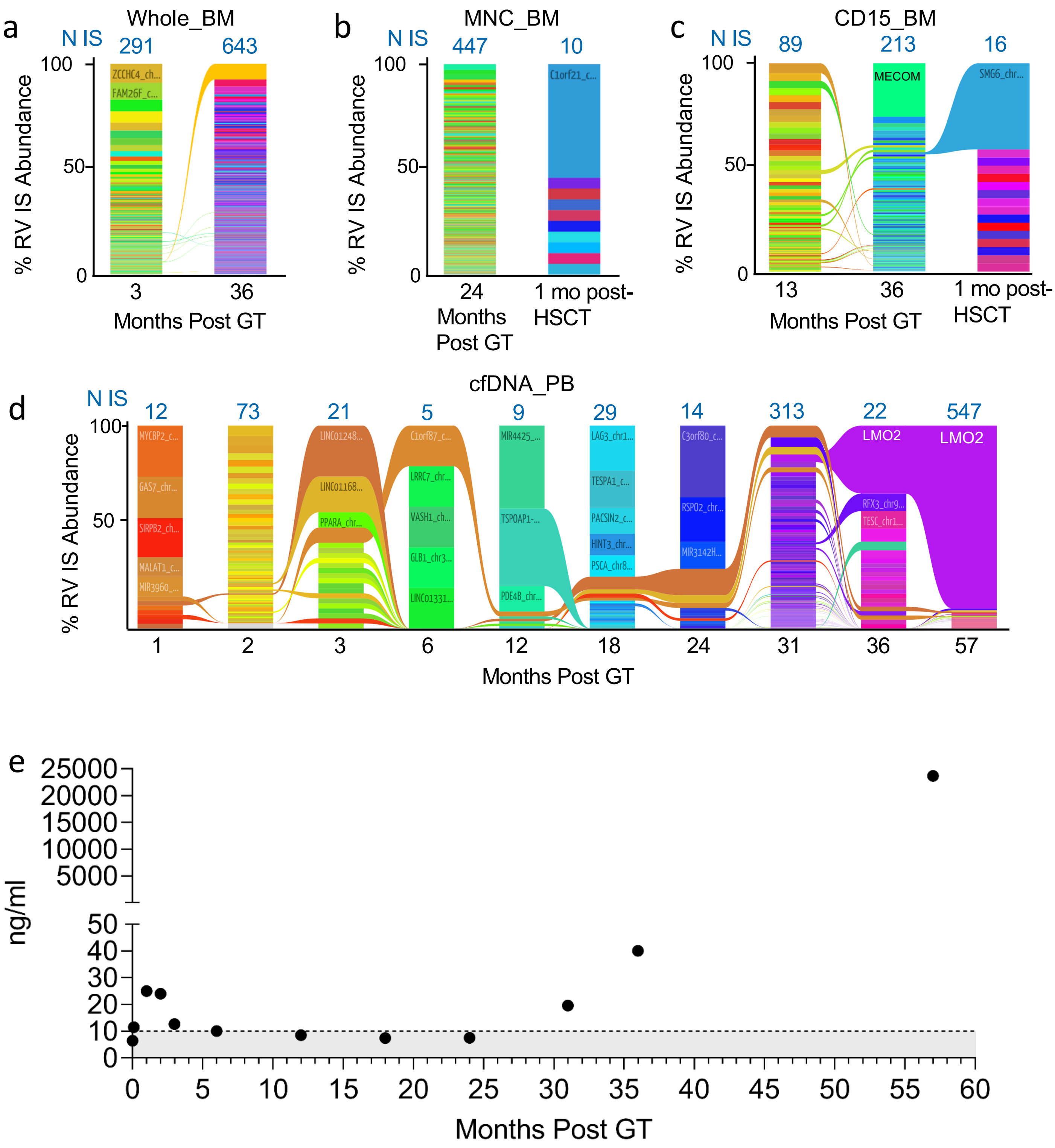

Supplementary Figure 2

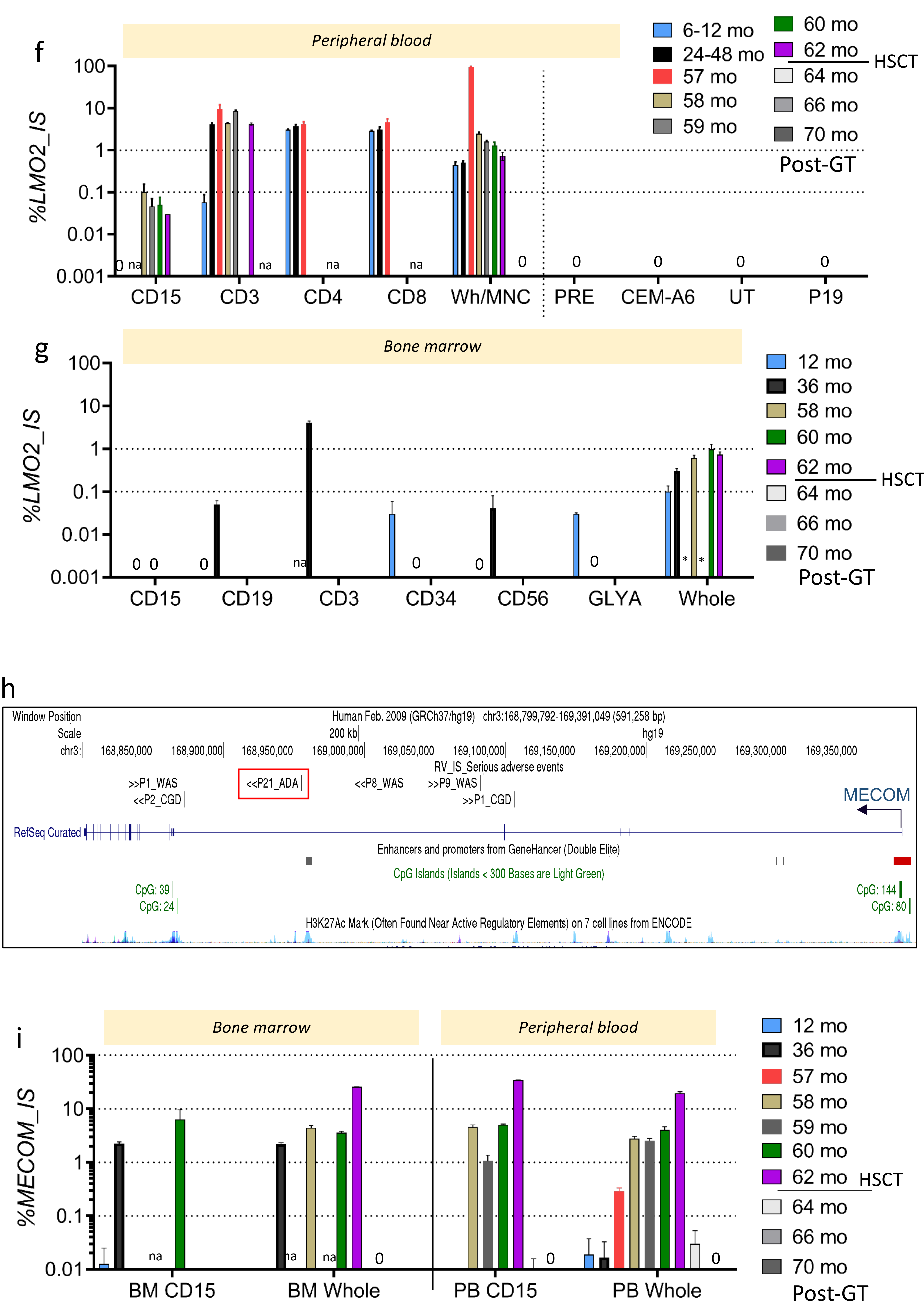

Supplementary Figure 2: Distribution of vector integrations into *LMO2* and *MECOM* genes in patients that developed adverse events. a-d) Stacked bar plots showing the abundance of γRV IS retrieved (months, x-axis) in BM-derived whole blood (a), MNC (b) and CD15 (c) cells as well as in PB-derived cfDNA (d) collected at different time points post-GT. In each column, each γRV IS is represented by different colors, whose height is proportional with the number of genomes retrieved for that IS over the total and for each specific time point (%IS Abundance, y-axis). Ribbons connect γRV IS tracked among consecutive time points. The number of unique IS retrieved from each specific time points is indicated in blue above each column; e) cfDNA recovered over time per ml of blood plasma (months post GT, x-axis) for P21 ADA patient; f) Quantification of the relative abundance of the γRV IS identified in T-ALL clone (P21) and close to *LMO2,* measured overtime by droplet-digital PCR in peripheral blood (g) and bone marrow-derived (g) cells as indicated. LMO2 clone abundance was particularly evident in the PB lymphoid compartment (CD3+, CD4+ and CD8+ cells), reaching 3% levels since the first year post GT and remaining stable until the leukemia onset (4.4% average), thus confirming the results obtained from IS analyses. Although at lower levels, the same LMO2 IS was also detected in CD34+ and CD56+ cells purified from the patient's BM at 12 months (0.03% and 0.03% respectively); h) Genomic view of γRV IS in *MECOM* gene and retrieved from patients treated in other clinical trials that developed serious adverse events as a consequence of vector-driven insertional mutagenesis. γRV IS retrieved from P21_ADA patient (highlighted in a red box) was not associated to adverse event. Chromosome number, genomic coordinates and scale are indicated on top of each panel. Black lines indicate the position of the indicated γRV IS, black arrow indicate vector orientation. Patient_ID and disease are also indicated. *MECOM* genomic structure is indicated by blue bar and lines: blue boxes and vertical bars indicate exons; blue arrow indicate the start site and orientation of transcription. Gene regulatory regions such as CpG islands, Enhancer and Promoter sequence and histone methylation marks are also indicated by the specific USCS genomic track (in GeneHancer track red box referred to promoter, grey box to enhancer). i) Quantification of the relative abundance of the γRV IS within *MECOM* gene in P21 measured overtime by droplet-digital PCR in bone marrow- (left) and peripheral blood- (right) derived cells as indicated. For *LMO2* and *MECOM* ddPCR, primer and probes specific for the IS were adopted and abundance were normalized on the total amount of GAPDH, ddPCR sensitivity <0.01%. In f, g and i data are represented as mean values of technical replicate values +/- SEM (N=3). Source data are provided as a Supplementary Data Fig2.

Supplementary Figure 3

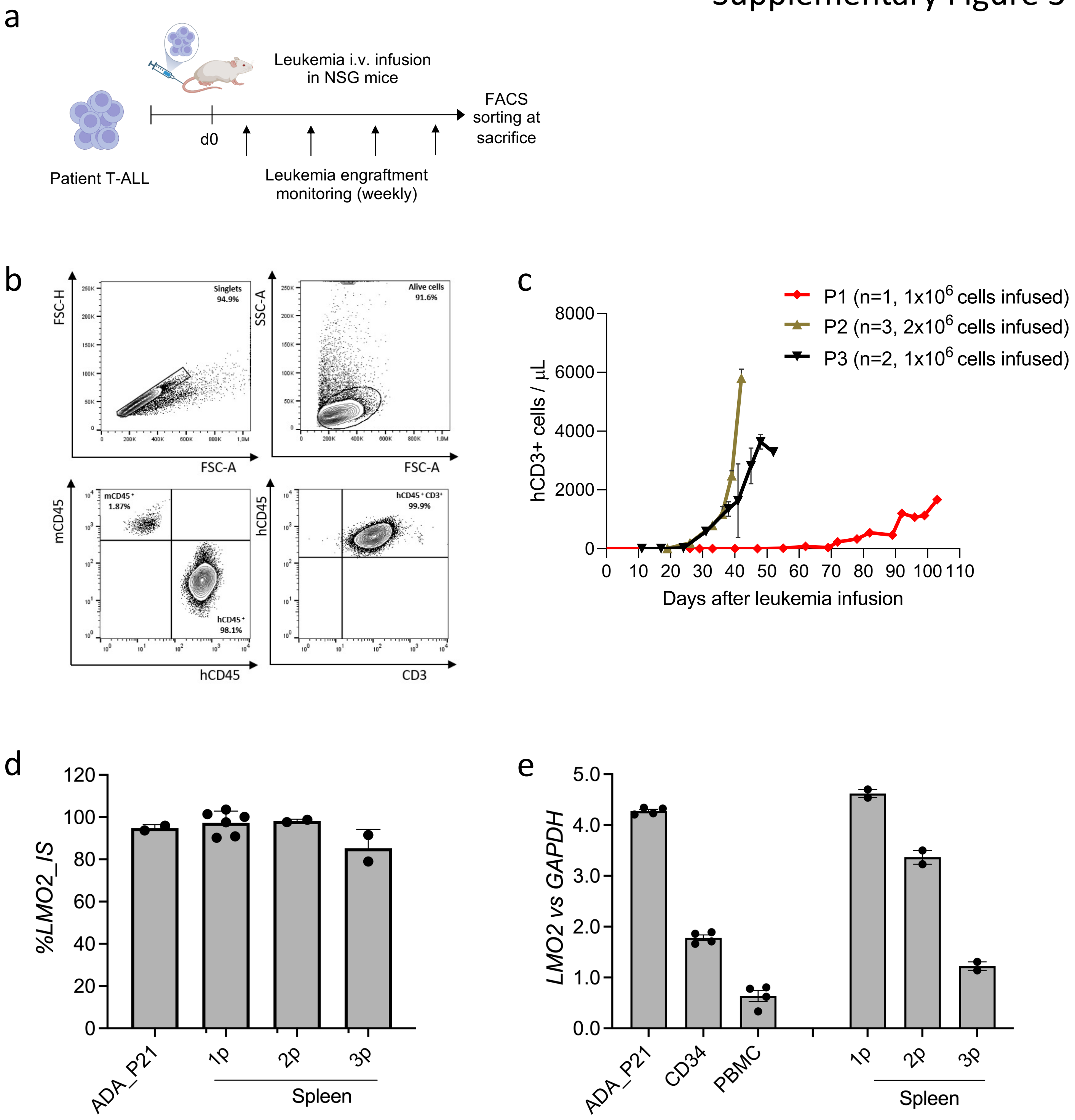

Supplementary Figure 3: Generation of Patient-Derived Xenografts (PDX) from P21 T-ALL, and analysis of their molecolar features. a) Schematic representation of PDX generation and analysis. As previously described, T-ALL cells from P21 were infused into non-irradiated 4-weeks-old NSG mice. Presence and absolute counts of human CD3+ cells in the peripheral blood of the animals were monitored weekly by retro-orbital bleeding and immunophenotypic flow cytometry analysis. Upon appearance of signs of suffering, mice were euthanized, and cells harvested from the spleen were analyzed for phenotype, vector integration and LMO2 expression. b) Surface phenotype of the infused leukemia in the animals harvested from mice after a first *in vivo* passage. c) Leukemia growth kinetics upon serial passaging, measured as absolute counts of human CD3+ cells in the peripheral blood. Shown are average values from the indicated number of animals, and whiskers display standard error mean. d) Percentage of leukemic cells bearing vector integrations in the primary sample and in cells harvested from the spleen of the animals at different serial passages (1p, 2p, 3p). T-ALL blast cells engrafted and expanded in serially transplanted patient-derived xenograft models (PDXs) maintaining the immune-phenotypical features and genetic marking of the parental cells; e) Relative expression of the *LMO2* transcript in leukemic cells from the primary sample and in cells harvested from the spleen of the animals at different serial passages. T-ALL cells engrafted and expanded in patient-derived xenograft models and revealed a progressive reduction in *LMO2*-expression over *in vivo* passages, suggesting for an evolution of oncogene addiction allowing T-ALL blasts to overcome the initially required overexpression from the initiating oncogenic transcription factors. These results are in line with those recently obtained in a regulated T-ALL mouse models where continuous *LMO2* expression is required for the maintenance and self-renewal of thymus resident pre-leukemic stem cells, but once established leukemia can relapse after *LMO2* withdrawal due to loss of tumor suppressor genes such as Ikaros1. In d and e, data are presented as mean values +/- SEM of biological replicate (N=2-4). Source data are provided as a Supplementary Data Fig3.

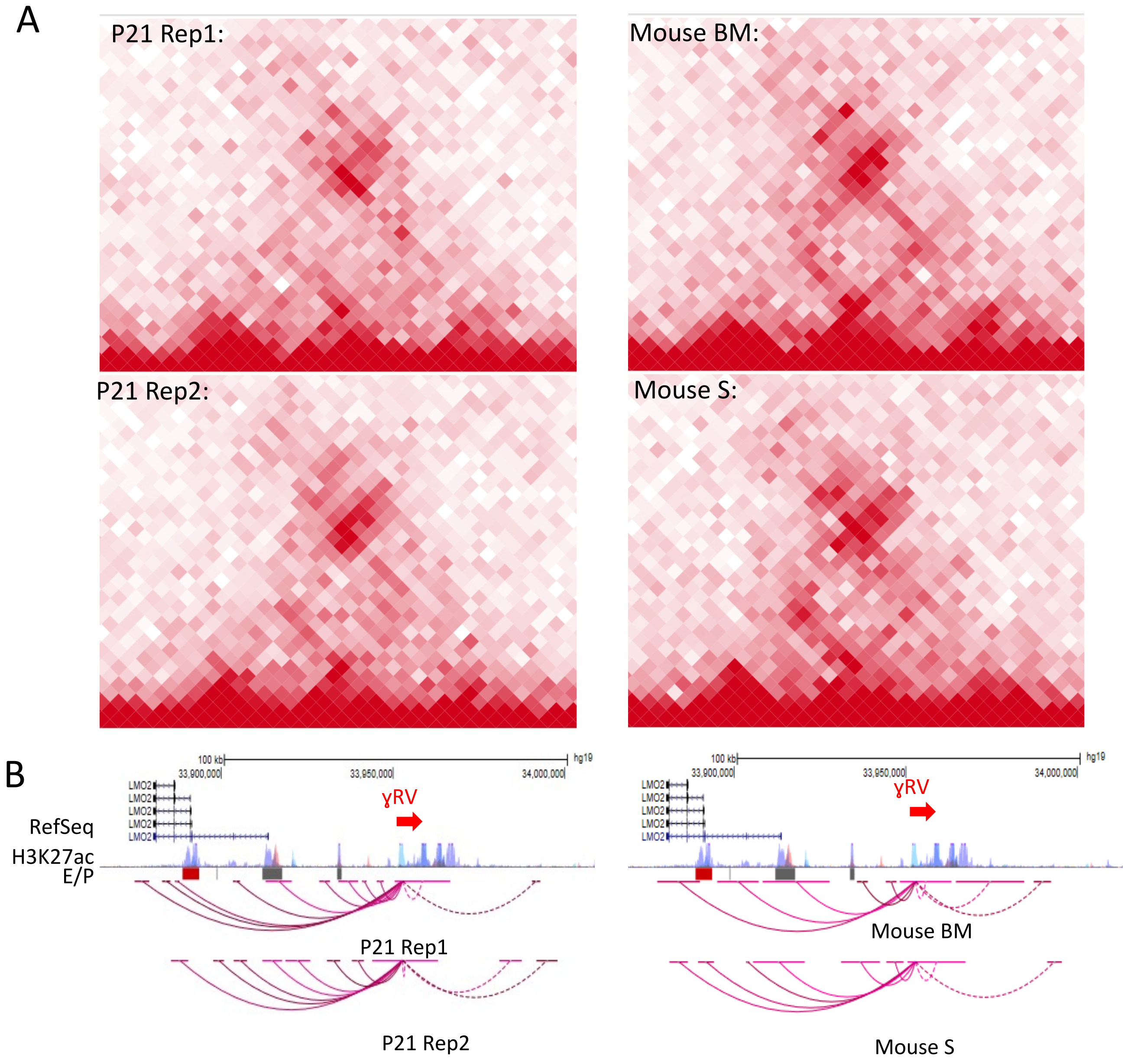

Supplementary Figure 4: ɣRV and host genome chromatin interactions at the LMO2 locus. a) Chromatin structure at the LMO2 locus: *in-situ* Hi-C was performed on blast cells collected from P21 ADA (P21 Rep1 and P21 Rep2) and on samples engrafted and expanded in PDX models (Mouse BM and Mouse S). Following bioinformatics analysis with Juicer software (https://github.com/aidenlab/juicer), 5Kb resolution Hi-C matrixes, describing physical contacts throughout the genome, were computed, and the interaction data used to identify topologically associated domains (TADs). In all samples the *LMO2* transcription unit and its associated regulatory elements were consistently contained within a single TAD of 135Kb. and from xenograft-derived BM and Spleen samples; b) We next extracted sequencing reads containing both γ-RV and human genomic sequences, defined as chimeric reads, and mapped their distribution on the genome. Chimeric reads mapping at distant locations from the IS, representing bona fide biologically relevant long-range interactions, were selected and to identify significant vector-host genome interactions, chimeric reads distant less than 1kb to each other were clustered to form interaction peaks (ITR). Strong interaction peaks stemming from the vector were confined within a 149Kb-window and displayed an asymmetric pattern of interaction, where most of the interactions pointed toward a genomic region upstream the vector IS and specifically to the *LMO2* gene where the enhancer and promoter of the gene as well as other regions enriched for active histone marks and TF-binding sites are located, thus physically linking the integrated vector sequence to the host genomic regulatory elements of *LMO2*. In the figure, LMO2 gene and gamma-RV integration site (IS) are shown RV-interaction peaks with the surrounding genome are indicated as arcs. Enhancer-promoters and active chromatin marks from K562 cells are shown. Source data are provided as a Supplementary Data Fig4.

a. Allele 1. PTEN, exon 7:chr10 (del/insertion of 6 nts, Pro231/Val403delInsHisAsp)

Reference protein

1 MTAIIKEIVS RNKRRYQEDG FDLDLTYIYP NIIAMGFPAE RLEGVYRNNI DDVVRFLDSK
61 HKNHYKIYNL CAERHYDTAK FNCRVAQYPF EDHNPPQLEL IKPFCEDLDQ WLSEDDNHVA
121 AIHCKAGKGR TGVMICAYLL HRGKFLKAQE ALDFYGEVRT RDKKGVTIPS QRRYVYYYSY
181 LLKNHLDYRP VALLFHKMMF ETIPMFSGGT CNPQFVVCQL KVKIYSSNSG **PTRREDKFMY**
241 **FEFPQPLPVC GDIKVEFFHK QNKMLKKDKM FHFWVNTFFI PGPEETSEKV ENGSLCDQEI**
301 **DSICSIERAD NDKEYLVLTL TKNDLDKANK DKANRYFSPN FKVKLYFTKT VEEPSNPEAS**
361 **SSTSVTPDVS DNEPDHYRYS DTTDSDPENE PFDEDQHTQI TKV***

Protein predicted from variant coding sequence

1 MTAIIKEIVS RNKRRYQEDG FDLDLTYIYP NIIAMGFPAE RLEGVYRNNI DDVVRFLDSK
61 HKNHYKIYNL CAERHYDTAK FNCRVAQYPF EDHNPPQLEL IKPFCEDLDQ WLSEDDNHVA
121 AIHCKAGKGR TGVMICAYLL HRGKFLKAQE ALDFYGEVRT RDKKGVTIPS QRRYVYYYSY
181 LLKNHLDYRP VALLFHKMMF ETIPMFSGGT CNPQFVVCQL KVKIYSSNSG **HD***

b. Allele 2. PTEN, exon 7: chr10 (Glu235/Val403del-Arg)

Reference protein

1 MTAIIKEIVS RNKRRYQEDG FDLDLTYIYP NIIAMGFPAE RLEGVYRNNI DDVVRFLDSK
61 HKNHYKIYNL CAERHYDTAK FNCRVAQYPF EDHNPPQLEL IKPFCEDLDQ WLSEDDNHVA
121 AIHCKAGKGR TGVMICAYLL HRGKFLKAQE ALDFYGEVRT RDKKGVTIPS QRRYVYYYSY
181 LLKNHLDYRP VALLFHKMMF ETIPMFSGGT CNPQFVVCQL KVKIYSSNSG PTRR**EDKFMY**
241 **FEFPQPLPVC GDIKVEFFHK QNKMLKKDKM FHFWVNTFFI PGPEETSEKV ENGSLCDQEI**
301 **DSICSIERAD NDKEYLVLTL TKNDLDKANK DKANRYFSPN FKVKLYFTKT VEEPSNPEAS**
361 **SSTSVTPDVS DNEPDHYRYS DTTDSDPENE PFDEDQHTQI TKV***

Protein predicted from variant coding sequence

1 MTAIIKEIVS RNKRRYQEDG FDLDLTYIYP NIIAMGFPAE RLEGVYRNNI DDVVRFLDSK
61 HKNHYKIYNL CAERHYDTAK FNCRVAQYPF EDHNPPQLEL IKPFCEDLDQ WLSEDDNHVA
121 AIHCKAGKGR TGVMICAYLL HRGKFLKAQE ALDFYGEVRT RDKKGVTIPS QRRYVYYYSY
181 LLKNHLDYRP VALLFHKMMF ETIPMFSGGT CNPQFVVCQL KVKIYSSNSG PTRR**RKTSSC**
241 **TLSSLSRYLC VVISK***

Supplementary Figure 5: Predicted protein sequences of PTEN consequent to the presence of the identified somatic variations. WGS and Exome sequencing analyses identified 2 mutations in the Ex7 genomic sequence of PTEN gene. Both mutations led to missense and frameshift mutations in the predicted protein coding sequence causing a premature stop codon formation. The predicted sequence of the altered PTNE protein is indicated. For each mutation: the upper panel reported the PTEN wild-type sequence. In green are indicated those amino-acid residues that are lost as a result of the PTEN gene mutation. the lower panel reported the sequence of the mutated protein variant. In red are highlighted the amino acid residues that are introduced in the protein sequences as result of the indicated genomic mutations.

a) Predicted PTEN protein sequence consequent to the del/insertion of 5 nucleotides: the c.692_697delinsATGACT variant caused amino-acid deletion from Pro231 to Val403, and insertion of His and Asp residues before the occurrence of a STOP codon;
b) Predicted PTEN protein sequence consequent to the 2 nucleotides insertion (CG): the c.702_703insCG variant led to frameshift mutations of the protein sequence starting from Glu235 and the occurrence of a STOP codon at residue 255.

Supplementary Figure 6

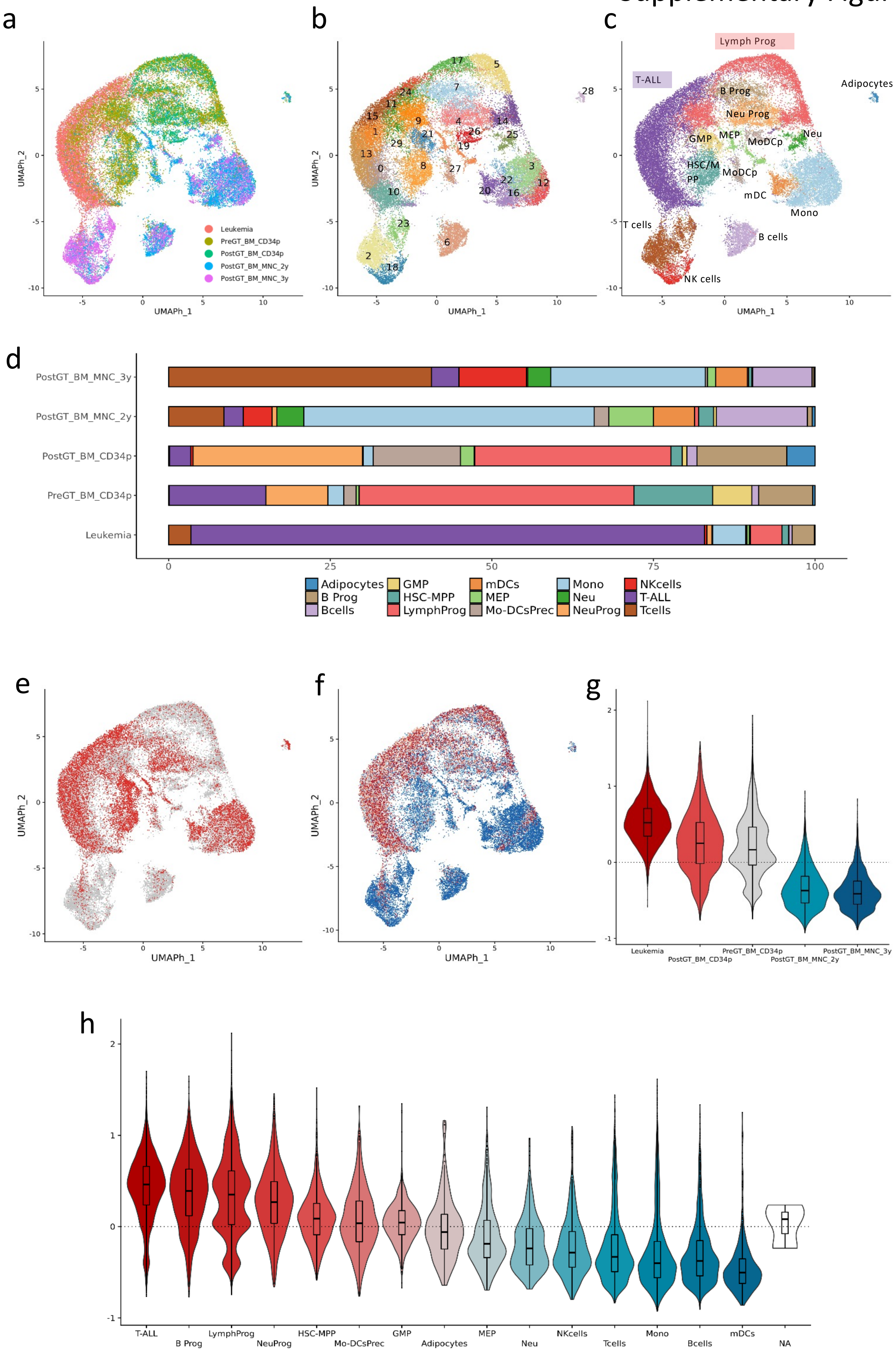

Supplementary Figure 6: Single cell analyses Single cell RNA sequencing (scRNAseq) was performed on BM cells at the time of T-ALL diagnosis, before gene therapy (CD34+ cells) and during the first 3 years after GT (CD34+ and mononuclear cells), up to 21 months before leukemia onset. a-c) UMAP plots showing cells coloured by clinical sample (a), unsupervised clustering at resolution 1.8 (b) and cell type annotation after manual inspection of marker genes (c); With respect to the pre-leukemic samples, T-ALL mostly occupied distinct clusters on the 2D-UMAP projection of the merged dataset and expressed typical genes associated with developing/proliferating T cells, including TCR/CD3, LCK, SOX4, TCF7, IL32, BCL11B, JARID2, CCND3, CDK6, MKI67; d) Stacked bar plots showing the relative distribution of cell types within each of the 5 clinical sample types, applying the cell type annotation shown in (c); the pre-GT CD34+ BM sample differed from the post-GT sample by a high proportion of lymphoid progenitors, some of which overlapped with the leukemia cluster; e) UMAP plot showing cells coloured by presence (red) or absence (grey) of the LMO2 transcript; *LMO2* was highly and homogenously expressed across all leukemia populations, but also in monocytes and dendritic cells and the majority of CD34+ cells, even before GT, making it an unreliable transcriptional marker to distinguish the leukemic clone from non-malignant progenitors. f) UMAP plot showing expression of the module score composed of the following genes, derived from bulk RNA sequencing: CDK6, SOX4, MYH9, KMT2D, MYB, DNM2, NF1 and KRAS that; g-h). Violin-Plots showing distribution of the module score from (F) according to clinical sample type (G) or cell type annotation (H), ordered by median value. The identified module score accurately distinguished leukemia from mononuclear BM cells sampled 2-3 years before leukemia diagnosis, arguing that the pre-leukemic *LMO2* clone detected at low frequency in CD3+ T cells (~4%) and whole BM (~0.5%) at the 3 years timepoint was not directly related to progression toward transformation. Instead, non-leukemic CD34+ BM populations (where the preleukemic LMO2 clone was absent) showed somewhat higher expression of the before-mentioned score, especially in some of the lymphoid progenitor populations prevalent in the pre-GT. Interestingly, these clusters express low/absent levels of endogenous *LMO2*, raising the hypothesis that its ectopic expression through insertional mutagenesis may have contributed to the transformation of such lymphoid lineage-committed progenitor populations, potentially enriched in ADA-SCID patients before GT. Source data are provided as a Supplementary Data Fig5.

Supplementary Figure 7

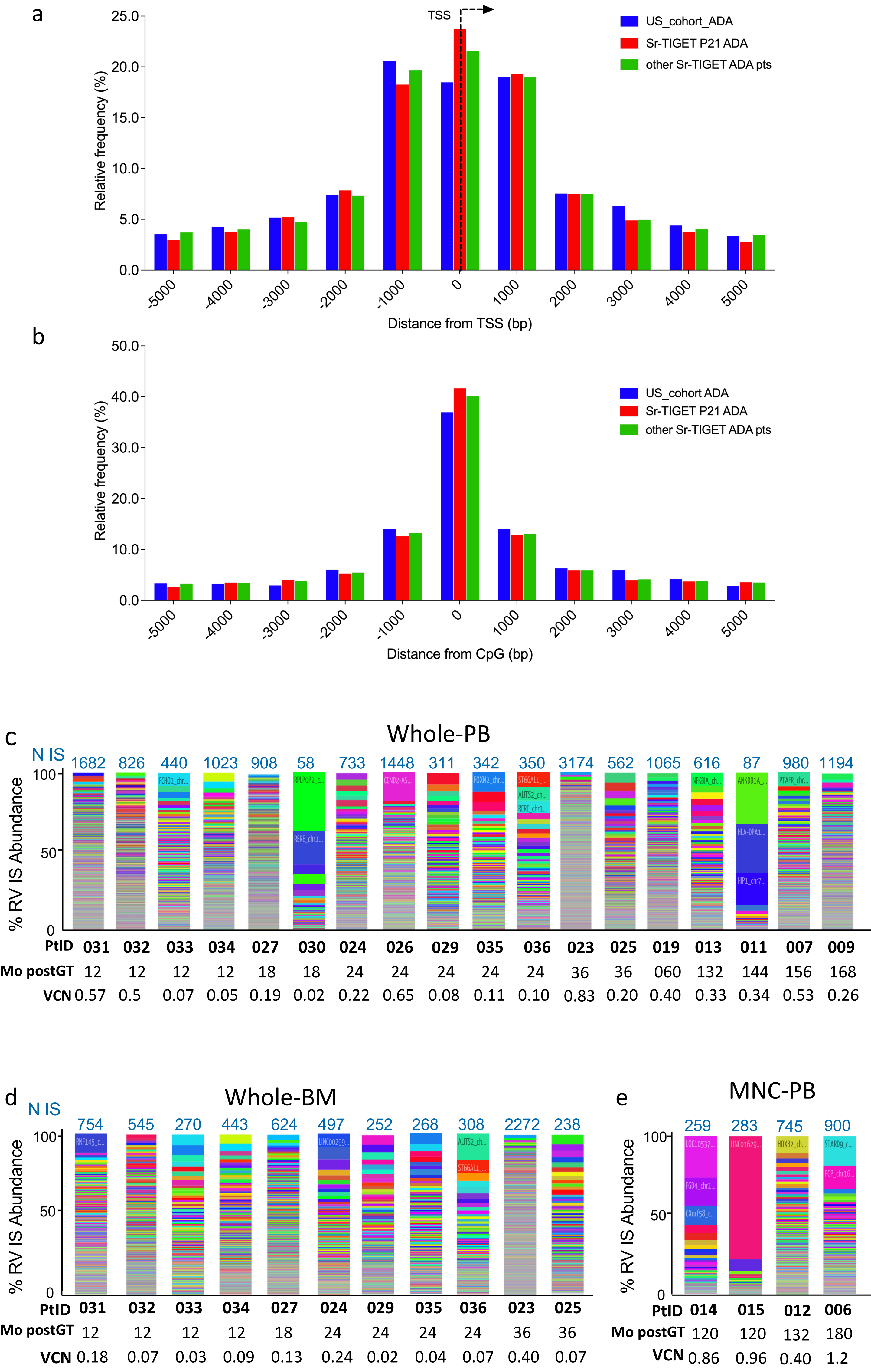

Supplementary Figure 7: Vector integrations collected from all other ADA patients treated by HSC-GT. a, b) Frequency distribution of γRV integrations around gene TSS (a) and CpG islands (b) of human RefSeq genes in SR-Tiget P21 ADA patient, all other SR-Tiget ADA patients and ADA patients from the US_cohort ADA, as indicated; the ISs were similarly found to be enriched within a 10 kb window on either side of the nearest TSS and close to CpG islands; c-e) Stacked bar plots showing the abundance of γRV IS retrieved (months, x-axis) from BM- and PB-derived whole and MNC cell population, as indicated. In each column, each γRV IS is represented by different colors, whose height is proportional with the number of genomes retrieved for that IS over the total and for each specific time point (%IS Abundance, y-axis). The number of unique IS retrieved from each specific time points is indicated in blue above each column, patientID, Timepoint post-GT (months) and VCN are indicated below the column. In Pt_11 and Pt_30 the relatively expanded clones occurred in samples were a limited number of insertions occurred, thus increasing the relative percent of each IS of the dataset. Otherwise, these expansion events could rather be the result of a physiological response of the immune system towards a specific antigen.

Supplementary Figure 8

a

SR-Tiget P21 ADA patient – GO Biological process

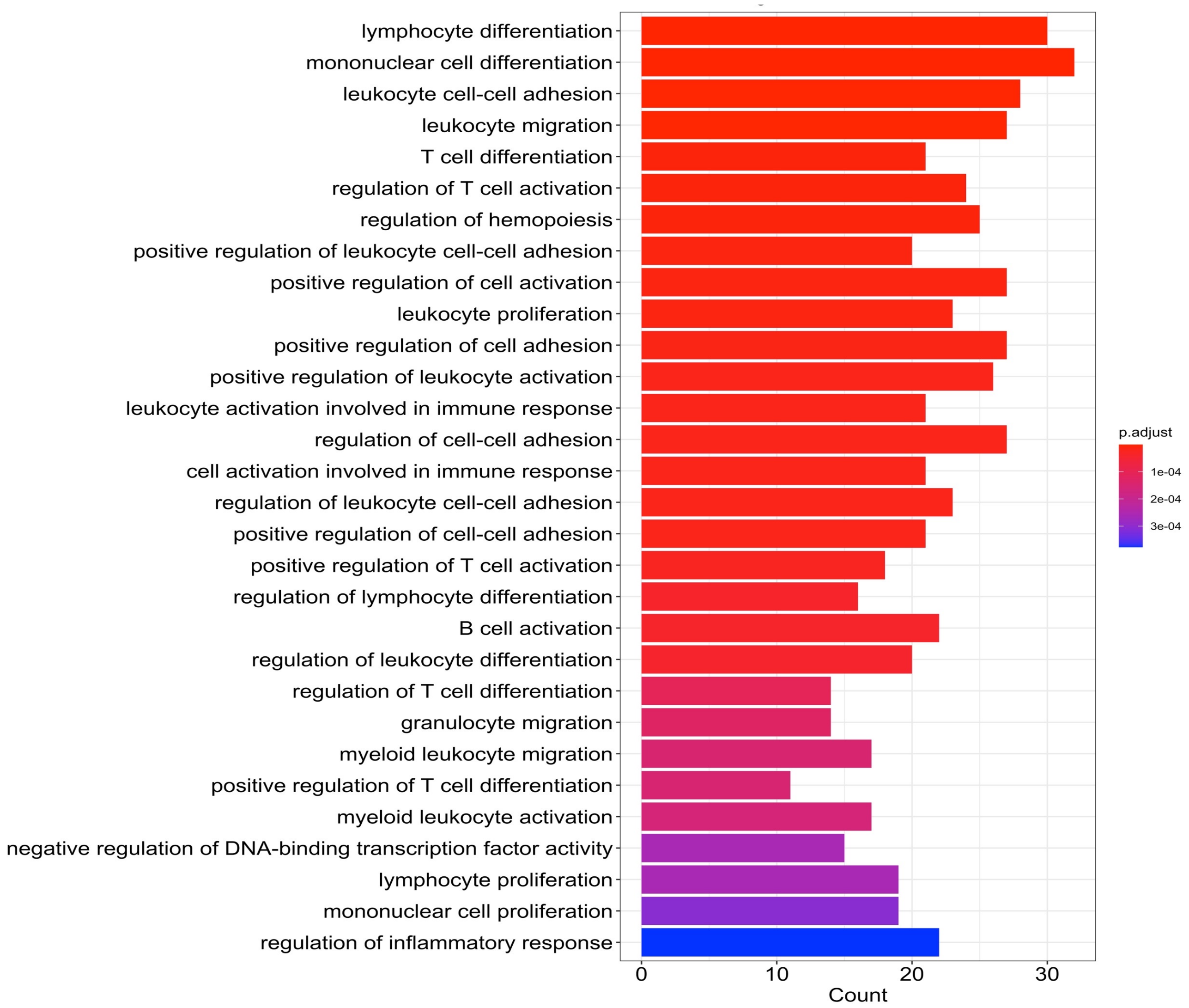

b

SR-Tiget cohort ADA patients – GO Biological process

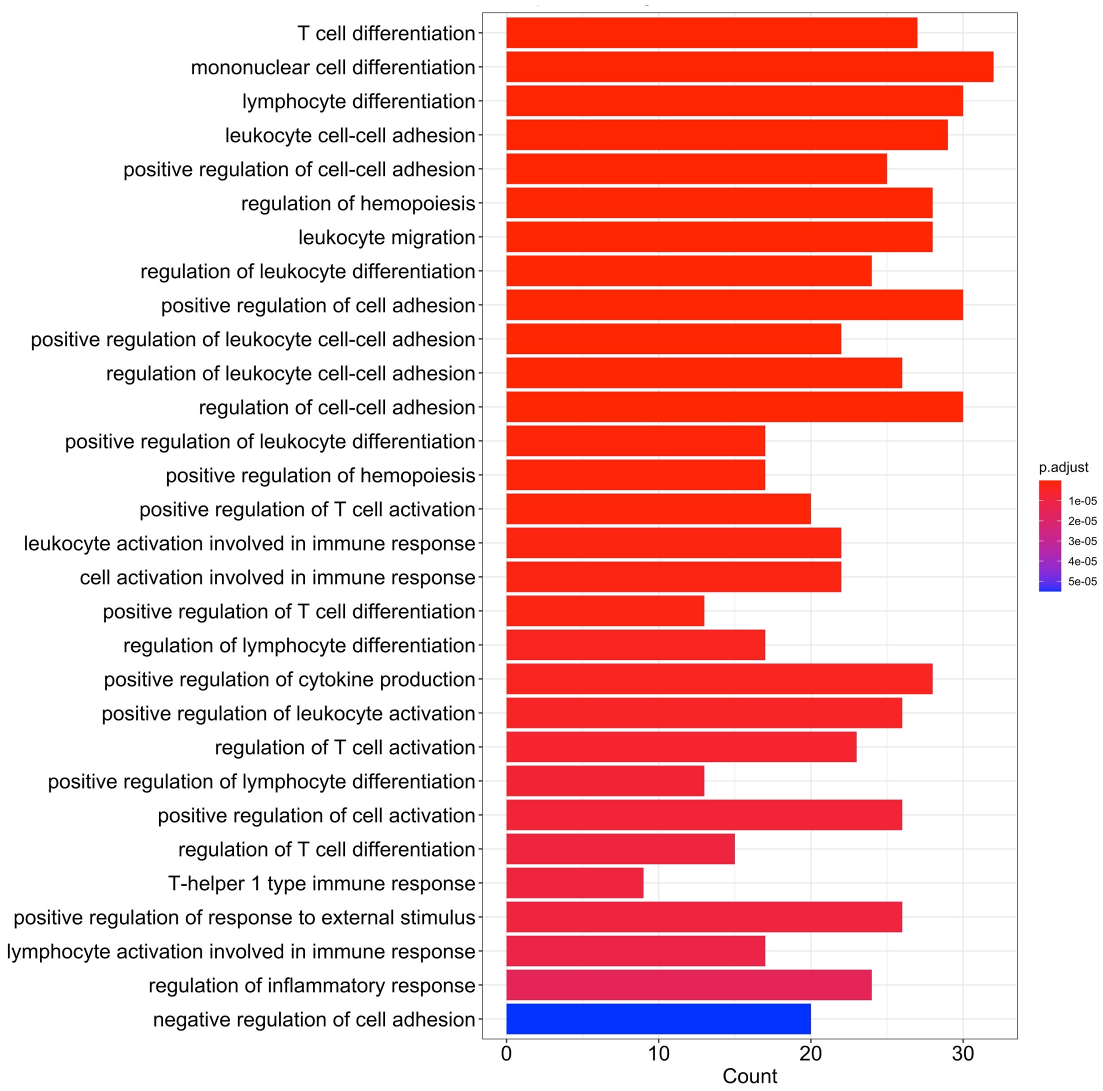

c

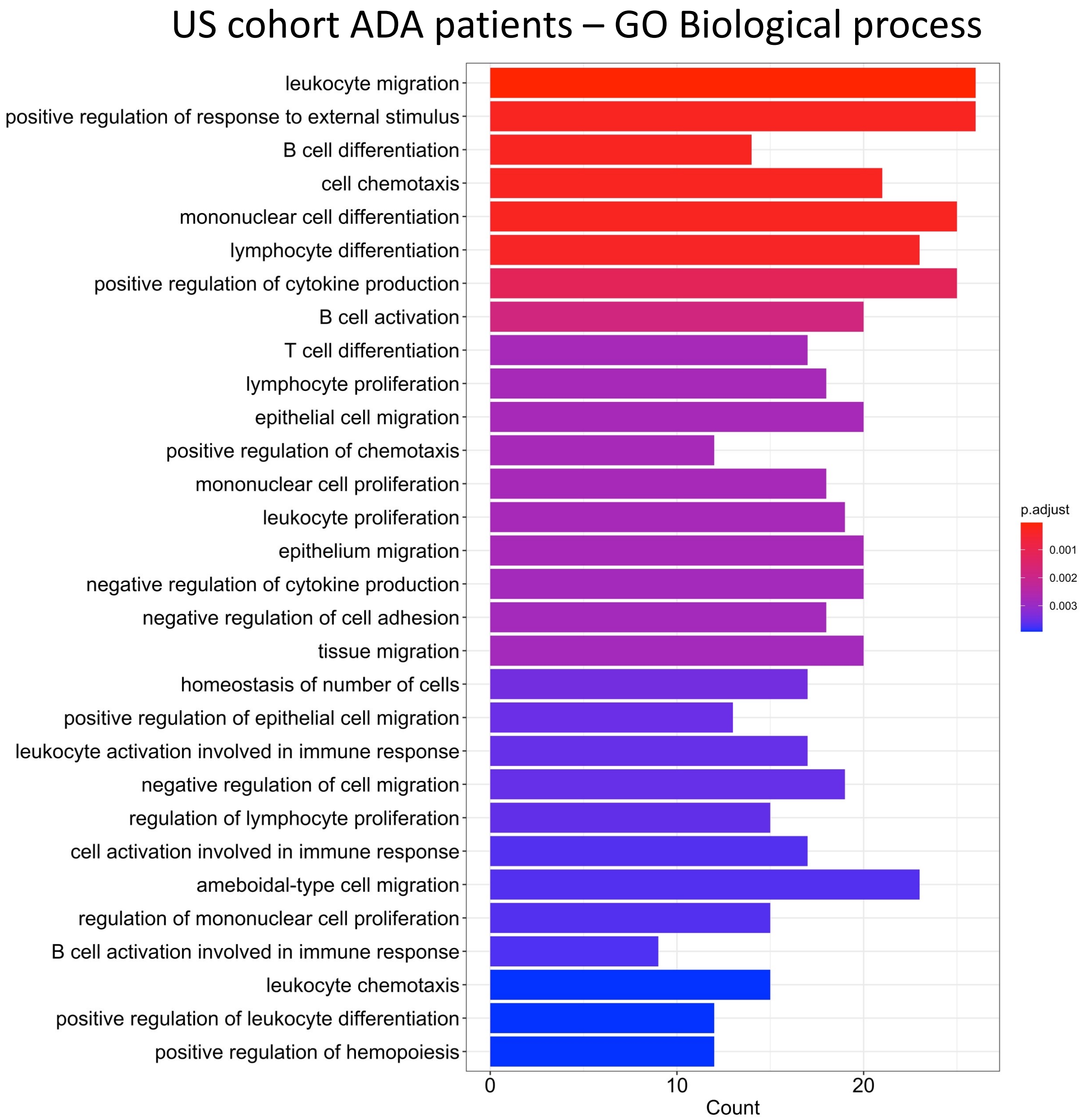
US cohort ADA patients – GO Biological process
leukocyte migration
positive regulation of response to external stimulus
B cell differentiation
cell chemotaxis
mononuclear cell differentiation
lymphocyte differentiation
positive regulation of cytokine production
B cell activation
T cell differentiation
lymphocyte proliferation
epithelial cell migration
positive regulation of chemotaxis
mononuclear cell proliferation
leukocyte proliferation
epithelium migration
negative regulation of cytokine production
negative regulation of cell adhesion
tissue migration
homeostasis of number of cells
positive regulation of epithelial cell migration
leukocyte activation involved in immune response
negative regulation of cell migration
regulation of lymphocyte proliferation
cell activation involved in immune response
ameboidal-type cell migration
regulation of mononuclear cell proliferation
B cell activation involved in immune response
leukocyte chemotaxis
positive regulation of leukocyte differentiation
positive regulation of hemopoiesis
0
10
20
Count
p.adjust
0.001
0.002
0.003

d

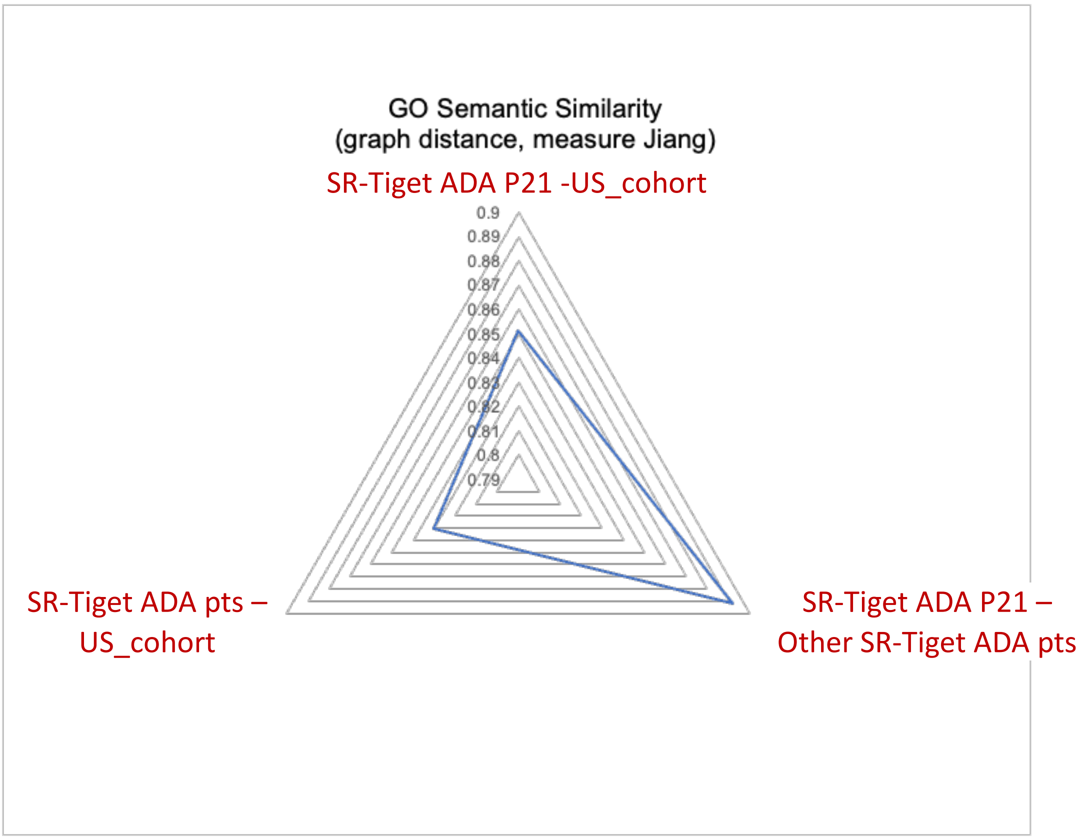
GO Semantic Similarity
(graph distance, measure Jiang)
SR-Tiget ADA P21 -US_cohort
0.9
0.89
0.88
0.87
0.86
0.85
0.84
0.83
0.82
0.81
0.8
0.79
SR-Tiget ADA pts – US_cohort
SR-Tiget ADA P21 – Other SR-Tiget ADA pts

Supplementary Figure 8: Gene ontology analyses. a-c) Gene ontology analysis for SR-Tiget P21 ADA patient, all other SR-Tiget ADA patients and ADA patients from the US_cohort showing the results for biological processes (BP), the only category resulted enriched in all datasets. First 500 genes were used to compute the GO enrichment. d) Triangular plot showing the GO semantic similarity distance between pairs of datasets (in vertexes) using the GO category biological processes (BP). A higher level of semantic correlation (0.89) was observed between the GO classes enriched in ADA_P21 and in the other ADA-SCID patients belonging to the same clinical cohort. Differently, a lower level of similarity was observed between ADA-P21 compared to the US cohort of patients (0.83).

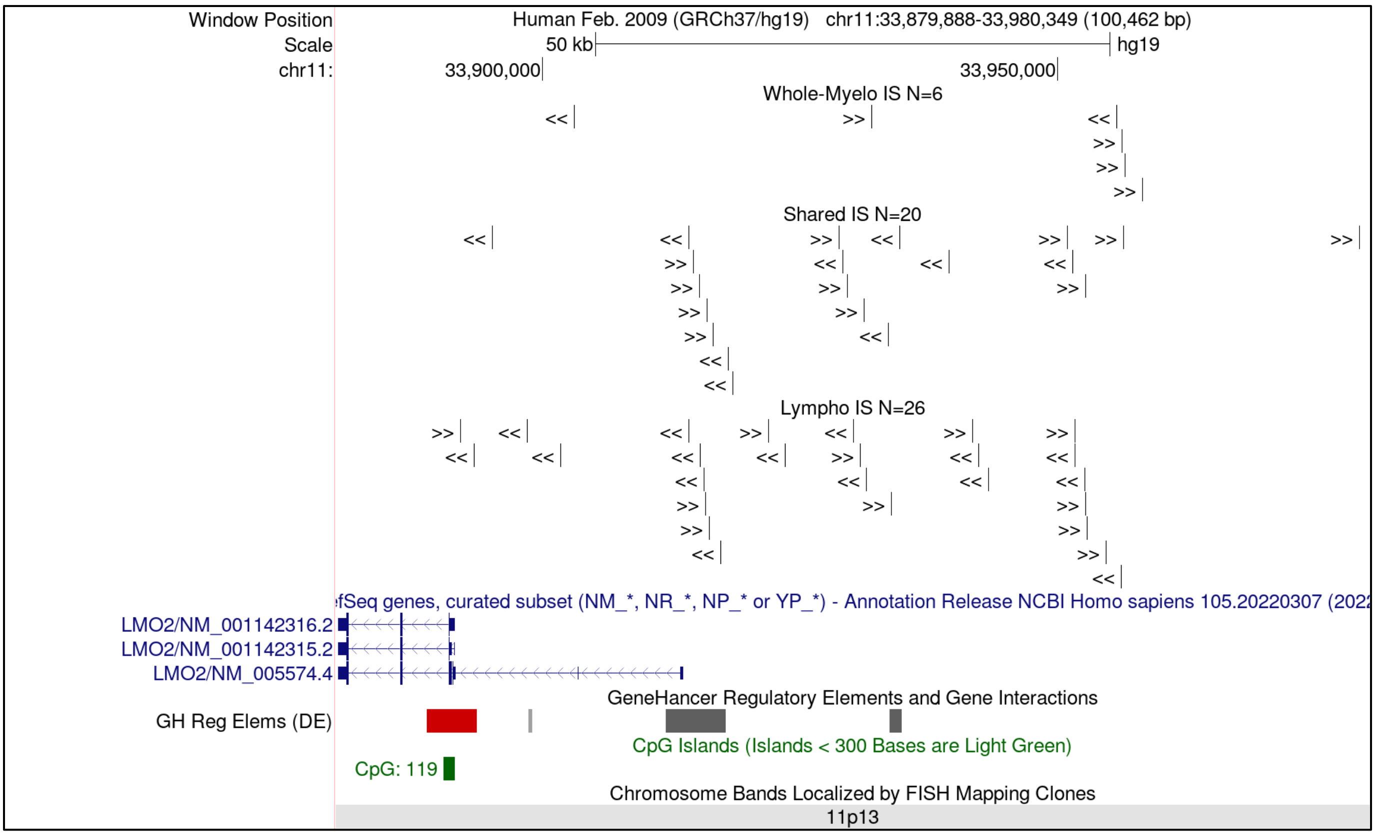

Supplementary Figure 9: Distribution of vector integrations into *LMO2* and *MECOM* genes in patients that developed adverse events. Genomic view of γRV IS close/in *LMO2* gene from ADA P21 samples collected at different time points (N=53). Indeed, γRV IS retrieved from T-ALL, Lymphoid (CD3, CD4 and CD8), Whole-Myeloid (highlighted in a red box) samples are indicated. Shared_IS referred to IS retrieved in both lymphoid and Whole-Myeloid samples. Chromosome number, genomic coordinates and scale are indicated at the top of the panel. Black lines indicate the position of the indicated γRV IS, black arrow indicate vector orientation. *LMO2* genomic structure is indicated by blue bar and lines: blue boxes and vertical bars indicate exons; blue arrow indicate the start site and orientation of transcription. Gene regulatory regions such as CpG islands, Enhancer and Promoter sequence and histone methylation marks are also indicated by the specific track (red box referred to promoter, grey box to enhancer).

Supplementary Table 1. γRV Integration Sites retrieved from P21

SubjectID	Tissue	Sample type	Time point (Mo)	N IS
P21 N= 10332	BM	CD15	13	89
	BM	CD15	36	213
	BM	CD15	1-HSCT	16
	BM	CD19	13	517
	BM	CD19	36	804
	BM	CD3	36	1928
	BM	MNC	24	447
	BM	MNC	1-HSCT	10
	BM	Whole	3	291
	BM	Whole	36	643
	PB	CD14	56	253
	PB	CD15	6	383
	PB	CD15	1-HSCT	5
	PB	CD3	6	2523
	PB	CD3	36	2078
	PB	CD4	12	35
	PB	CD4	18	253
	PB	CD4	24	2108
	PB	CD8	12	26
	PB	cfDNA	1	12
	PB	cfDNA	2	73
	PB	cfDNA	3	21
	PB	cfDNA	6	5
	PB	cfDNA	12	9
	PB	cfDNA	18	29
	PB	cfDNA	24	14
	PB	cfDNA	31	313
	PB	cfDNA	36	22
	PB	cfDNA	56	547
	PB	MNC	36	1937
	PB	MNC	56	428
	PB	MNC	1-HSCT	14
	PB	Whole	12	815
	PB	Whole	18	686
	PB	Whole	36	943
P04	PB	CD4	18	36

SubjectID	Tissue	Sample type	Time point (Mo)	N IS
P06 N=1590	PB	MNC	134	847
	PB	MNC	146	803
	PB	MNC	180	900
P07 N=2458	PB	MNC	49	1780
	PB	MNC	86	706
	PB	Whole	156	980
P09 N=4006	PB	MNC	86	1625
	PB	MNC	134	2637
	PB	PHA	132	222
	PB	Whole	168	1194
P11 N=187	PB	MNC	86	117
	PB	Whole	144	87
	PB	MNC	182	74
P12 N=1090	PB	MNC	98	596
	PB	MNC	132	745
P13 N=2503	PB	MNC	98	1905
	PB	Whole	132	616
	PB	MNC	182	1105
P14 N=448	PB	MNC	86	234
	PB	MNC	120	259
	PB	MNC	170	170
P15	PB	MNC	120	283
P19 N=2679	BM	MNC	36	501
	BM	Whole	60	967
	PB	PHA	37	1496
	PB	Whole	60	1065
P23 N=9146	BM	Whole	36	2272
	BM	MNC	49	448
	BM	MNC	61	1479
	PB	Whole	36	3174
	PB	MNC	49	3168
	PB	MNC	61	4309
P24 N=2494	BM	Whole	24	495
	BM	MNC	61	952
	PB	Whole	24	733
	PB	MNC	49	1465
	PB	MNC	61	901

SubjectID	Tissue	Sample type	Time point (Mo)	N IS
P25<br>N=2391	BM	Whole	36	240
	BM	MNC	61	615
	PB	Whole	36	562
	PB	MNC	49	1004
	PB	MNC	61	1136
P26<br>N=3450	BM	MNC	49	509
	BM	MNC	61	1109
	PB	Whole	24	1448
	PB	MNC	49	1117
	PB	MNC	61	1770
P27<br>N=2451	BM	Whole	18	624
	BM	MNC	36	848
	PB	Whole	18	908
	PB	MNC	36	1302
P29<br>N=1411	BM	Whole	24	252
	BM	MNC	36	198
	PB	Whole	24	311
	PB	MNC	36	202
	PB	MNC	43	784
	PB	MNC	49	471
P30<br>N=236	BM	MNC	49	72
	PB	Whole	18	58
	PB	MNC	36	115
	PB	MNC	49	90
P31<br>N=4100	BM	Whole	12	754
	BM	MNC	36	1824
	PB	Whole	12	1682
	PB	MNC	36	2319
P32<br>N=3616	BM	Whole	12	545
	BM	MNC	25	697
	BM	MNC	36	783
	PB	Whole	12	826
	PB	MNC	12	1550
	PB	MNC	12	1752
P33<br>N=1201	BM	Whole	12	270
	BM	MNC	36	358
	PB	Whole	12	440
	PB	MNC	36	614

SubjectID	Tissue	Sample type	Time point (Mo)	N IS
P34 N=2550	BM	Whole	12	443
	BM	MNC	24	440
	BM	MNC	36	713
	PB	Whole	12	1023
	PB	MNC	24	1130
	PB	MNC	36	934
P35 N=2118	BM	Whole	24	268
	BM	MNC	36	320
	PB	Whole	24	342
	PB	MNC	49	1575
P36 N=1873	BM	Whole	24	308
	BM	MNC	36	189
	BM	MNC	49	527
	PB	Whole	24	350
	PB	MNC	36	640
	PB	MNC	55	627

SubjectID: patient identifier, N means the total number of IS retrieved from the indicated patient.

Mo, months

Supplementary Table 2. Top abundant γRV Integration Sites retrieved from P21ADA patient in PB-derived samples

CD4_PB						
TOP	12 months		18 months		24 months	
	Gene ID	%Ab	Gene ID	%Ab	Gene ID	%Ab
1	OR10G7	7.5	LMO2	3.9	LMO2	3.8
2	ADGRG5	7.5	LINC01475	3.3	CLU	1.1
3	LINC00841	6.0	CTSS	3.3	YARS2	0.9
4	MIR4660	6.0	UNQ6494	1.6	YY1	0.9
5	ODF2	6.0	SMARCA2	1.4	SLC5A10	0.9
6	LMO2	6.0	LINC00501	1.4	SOCS2	0.8
7	SOCS2	4.5	SUN5	1.2	IRAK2	0.8
8	MECOM	4.4	SMPD3	1.2	SMARCA2	0.7
9	UBASH3B	3.1	DACH1	1.2	IL2RA	0.6
10	TTTY12	3.0	BATF	1.2	LMO2	0.6

Whole_PB						
TOP	12 months		18 months		36 months	
	Gene ID	%Ab	Gene ID	%Ab	Gene ID	%Ab
1	PTPRD	2.4	LMO2	2.0	MECOM	4.7
2	FAM135B	2.2	FAM135B	1.8	LMO2	3.4
3	DCT	2.1	LFNG	1.3	YY1	1.3
4	STARD13	1.8	DCT	1.1	CDK2AP1	1.2
5	LMO2	1.4	SLC25A38	1.0	YARS2	1.0
6	LFNG	1.3	PTPRD	1.0	LOC101928659	0.9
7	NONO	1.0	INPP5A	1.0	LMO2	0.9
8	INPP5A	0.9	BROX	0.8	LINC01248	0.6
9	SLC25A38	0.9	YARS2	0.8	SV2B	0.6
10	PLAC8	0.7	CLU	0.7	MEI1	0.6

MNC_PB						
TOP	36 months		57 months		1-HSCT	
	Gene ID	%Ab	Gene ID	%Ab	Gene ID	%Ab
1	LMO2	4.1	LMO2	90.8	IRAK2	33.9
2	YARS2	1.4	LINC01248	0.8	AGTRAP	31.0
3	YY1	1.2	LINC01168	0.7	PCSK7	2.9
4	CDK2AP1	1.0	MECOM	0.5	GRM6	2.9
5	LFNG	0.9	C1orf87	0.2	LMO7	2.9
6	MECOM	0.9	CACNA1C	0.1	MIR4487	2.9
7	CLU	0.7	CUX1	0.1	MIR8060	2.9
8	STX8	0.6	FBXW11	0.1	HGSNAT	2.9
9	SUSD1	0.6	MKLN1-AS	0.1	ROR2	2.9
10	SLC5A10	0.6	FLJ42351	0.1	LINC01947	2.9

cfDNA_PB										
TOP	1 month		2 months		3 months		6 months		12 months	
	Gene ID	%Ab	Gene ID	%Ab	Gene ID	%Ab	Gene ID	%Ab	Gene ID	%Ab
1	MYCBP2	25.2	RPL23AP87	5.2	LINC01248	25.1	C1orf87	20.0	MIR4425	40.9
2	GAS7	20.4	ACTL7B	4.6	LINC01168	17.6	LRRC7	20.0	TSPOAP1-AS1	38.1
3	SIRPB2	19.3	UBE2W	3.9	PPARA	7.6	VASH1	20.0	PDE4B	12.6
4	MALAT1	9.7	MIR146B	3.3	C1orf87	7.5	GLB1	20.0	C1orf87	2.4
5	MIR3960	9.6	EFHB	3.2	GLYAT	2.5	LINC01331	20.0	CXXC5	1.2
6	C1orf87	2.3	DENND1B	3.2	NLRP3	2.5			LINC01248	1.2
7	LINC01248	2.3	ANAPC7	2.6	LINC00448	2.5			LINC01559	1.2
8	STN1	2.3	IKZF1	2.6	TBX18	2.5			DUSP7	1.2
9	ABCD3	2.3	LCP1	2.6	XKR9	2.5			ASTN2	1.2
10	TRIM42	2.3	KSR1	2.6	DUSP7	2.5				

cfDNA_PB										
TOP	18 months		24 months		31 months		36 months		57 months	
	Gene ID	%Ab	Gene ID	%Ab	Gene ID	%Ab	Gene ID	%Ab	Gene ID	%Ab
1	LAG3	22.3	C3orf80	35.2	LINC01248	5.8	LMO2	33.5	LMO2	90.1
2	TESPA1	18.2	RSPO2	22.0	MECOM	4.7	RFX3	8.6	MECOM	0.9
3	PACSIN2	12.8	MIR3142HG	13.3	LINC01168	3.7	TESC	8.6	LINC01168	0.7
4	HINT3	10.6	LINC01248	13.0	LMO2	3.7	LMO2	6.4	LINC01248	0.7
5	PSCA	10.5	LINC01168	3.8	SIGLEC12	2.6	CXXC5	4.3	SEC13	0.3
6	LINC01248	6.2	C1orf87	2.5	C1orf87	2.4	GNG4	4.3	LINC01813	0.2
7	LINC01168	2.1	FHIT	1.3	STXBP4	2.1	GNA15	2.1	EMCN	0.2
8	TRIM42	2.1	ANKS1B	1.3	SSBP4	2.1	DR1	2.1	HYDIN	0.2
9	C1orf87	1.4	UNC79	1.3	CELA1	1.9	CLINT1	2.1	LINC01031	0.2
10	CORO1B	0.7	CHST9	1.3	HLA-DOA	1.9	RREB1	2.1	DHFRP3	0.2

* γRV IS of T-ALL clone (Figure 2A) is indicated in orange
* γRV IS within MECOM (Suppl. Figure 2H) is indicated in green

Supplementary Table 3. Top abundant γRV Integration Sites retrieved from P21ADA patient in BM-derived samples

Whole_BM				
TOP	3 months		36 months	
	Gene ID	%Ab	Gene ID	%Ab
1	ZCCHC4	9.0	MECOM	7.4
2	FAM26F	8.0	LMO2	3.4
3	DOCK9	5.5	ZNF414	2.4
4	DYM	5.4	RBM39	1.3
5	RHOF	3.7	CDK2AP1	1.0
6	MALAT1	3.6	ADGRG1	1.0
7	WASF2	3.3	LOC10050…	0.9
8	CYTL1	2.9	SMG6	0.8
9	TBC1D7	2.5	YARS2	0.8
10	LINC01014	2.0	ZNF815P	0.7

MNC_BM				
TOP	24 months		1-HSCT	
	Gene ID	%Ab	Gene ID	%Ab
1	LMO2	2.7	C1orf21	54
2	FAM135B	2.3	ZMYND8	5.1
3	RBM39	2.3	ELAVL4	5.1
4	MECOM	1.3	AGO2	5.1
5	CDK2AP1	1.2	SYK	5.1
6	EPGN	1.1	MFGE8	5.1
7	BCAT1	1.0	DCLK1	5.1
8	LOC1005..	1.0	HAUS1	5.1
9	CHSY1	0.9	ABI3BP	5.1
10	TNIP1	0.8	IPO11	5.1

CD15_BM						
TOP	13 months		36 months		1-HSCT	
	Gene ID	%Ab	Gene ID	%Ab	Gene ID	%Ab
1	TMEM265	4.7	MECOM	25.7	SMG6	41.3
2	SV2B	3.7	LOC10050..	3.1	MSI1	3.9
3	NDUFV2	3.4	HTR1F	1.8	C3orf67	3.9
4	RAB27B	3.4	MSI2	1.7	PEX13	3.9
5	RFX3	3.4	TSPOAP1-AS1	1.7	PTCH1	3.9
6	SYNE3	3.4	TSPAN32	1.6	DNMT3A	3.9
7	INPP4B	3.1	SETDB2	1.4	GRM8	3.9
8	SSBP4	3.1	DR1	1.2	C4BPA	3.9
9	ANO10	2.7	LINC00299	1.1	NPAS3	3.9
10	LINC01139	2.7	STX8	1.1	PRPSAP2	3.9

* γRV IS of T-ALL clone (Figure 2A) is indicated in orange
* γRV IS within MECOM (Suppl. Figure 2H) is indicated in green

Supplementary Table 4. Mutations and rearrangements identified in the T-ALL clone from Whole Genome Sequencing (WGS), in which we detected structural variants (SV) deletions, inversions, break-ends, copy number variants (CNV), and from RNA-seq, in which we identified fusion transcripts, and from hematopoietic exome sequencing and Whole Exome Sequencing (WES).

Structural Variant (SV) deletions					
**Chromosome**	**Start Deletion**	**End Deletion**	**Gene Start**	**Gene End**	**Gene**
chr1	47231899	47313727	47216290	47232220	STIL
chr14	22053055	22528528	22052514	22053056	TRAV21
chr6	62921177	109525093	NA	NA	No. 327
chr7	38253416	38358510	38253380	38253429	TRGJ2
chr7	38253419	38362862	38253380	38253429	TRGJ2
chr7	142313665	142797122	142313184	142313666	TRBV4-1
chr7	142627399	142797457	142626649	142627399	TRBV20- 1
chr9	21770645	22004424	21802636	21867081	MTAP
chr9	21770645	22004424	21969805	21975157	CDKN2A
chr14	22148633	22499690	22147995	22148633	TRAV27
chr14	106257879	106373658	106257762	106258223	GHV3-22

SV Inversions (4.7 y)		
**Chromosome**	**Start Inversion**	**End Inversion**
chr1	96994606	196132737
chr12	1543405	51126181
chr20	12633960	28790180
chr22	27725611	39566211

Somatic SV Breakends		
**Chromosome**	**Breakpoint**	**In Gene**
chr3	29007481	RBMS3
chrX	23780110	AC131011.1 no RefSeq gene
chr5	487513	SLC9A3
chr6	103310175	NA
chr17	17476488	NA
chr19	46403212	NA

CNV				
**Chromosome**	**Start_Deletion**	**End_Deletion**	**Type**	**N. Genes**
chr6	62920929	109525221	Deletion	327
chr9	72852	21770208	LOH	207
chr9	22004926	40845855	LOH	267 (including CDKN2B)

**Mutations identified by exome sequencing**								
**Gene**	**Chr**	**Transcript**	**Nucleotide change**	**Amino acid change**	**oncoKB**	**Somatic**	**ACMG-AMP**	**gnomAD 2.1**
PTEN*	chr10	371953	c.692_697delinsATGACT	p.ProThrArg231Ter	LO	yes	Tier 1 - oncogenic	
PTEN*	chr10	371953	c.702_703insCG	p.Glu235ArgfsTer22	LO	yes	Tier 1 - oncogenic	

Two PTEN variants within the exon 7, a 6-nucleotide substitution and a dinucleotide insertion, were specifically found in the T-ALL blasts. Both mutations were predicted to cause premature termination of PTEN (Figure 4, Suppl. Figure 5). Hence, a loss of function of PTEN should occur in the leukemic cells altering the PI3K/AKT signaling pathway. These two loss-of-function *PTEN* mutations in compound heterozygosity were not previously described and likely played a role in clonal selection. These *PTEN* mutations were not detected in the patient's peripheral blood CD3+ and CD4+ T cells up to 3 years post-GT with a sensitivity of 10^{-4} (data not shown) suggesting that they were acquired at a later time by the pre-leukemic clone or that these mutations occurred in a non-circulating sub-clone confined to the thymus.

**Fusion transcripts by RNA-seq**		
**Chromosome**	**Region**	**Genes**
chr1	47213808-47319478	STIL-TAL1
chr3	134477447-135304600	EPHB1-CEP63
chr17	46027907-46384955	KANSL1-LRRC37A

RNA sequencing confirmed the overexpression of LMO2 in blast cells and the expression of the STIL-TAL1 and KANSL1-LRRC37A fusion transcripts. Furthermore, numerous highly expressed genes associated to T-ALL, like CDK6, SOX4, NEAT1, MYH9, EEF2, KMT2D, MYB, DNM2, NF1 and KRAS were found. Dominant alpha/beta T cell receptor chains were also detected to be highly expressed (TCRA and TCRB deletions: TRBV4-1 and TRBV20-1 and TRAV21, TRAV27 and TRAJ16), thus confirming the presence of a TCR clonotype in the leukemic cells.

Supplementary Table 5: List of germline-predisposing mutations identified in P21 that may have an impact on the tumorigenic outcome
It has been reported that approximately 8–10% of pediatric cancer patients harbor germline predisposing mutations (Jeffery M. Klco, er al. Nat Rev Cancer. 2021 doi:10.1038/s41568-020-00315-z). Hence, we look for the presence of germline mutations that could influence the risk for neoplastic transformation in P21. We specifically identified 21 non-silent germline mutations that are predicted impact protein function. Some of these mutations occurred in known tumor suppressor genes and transcription factors that have a role in cancer development. Among those, missense mutations were found in five genes (MSH6, ARID1A, CARD11, CBL and SRC) involved in T-cell development and previously found altered in lymphoid and myeloid leukemia.

Chr	Position hg38	Refseq	Varseq	VarType	RS ID	Zygosity	Allelic balance	Frequency	Impact	Genes	VarFunc	RNAseq	Putative Role	Notes
chr4	154587520	G	A	SNV	rs755117226	Het	0.55	8.0E-06	ns	FGA	P	No	-	Coagulation factor, Mutations can lead to congenital afibrinogenemia
chr19	19712062	G	A	SNV	rs114388860	Het	0.54	7.2E-05	ns	ZNF14	LP	Yes	-	Transcription factor
chr1	182794441	T	del	Del	rs761806358	Het	0.55	2.9E-04	sp	NPL	LP	No	-	Protein function involved in bowel disease
chr9	20820345	C	T	SNV	rs141620768	Het	0.51	2.0E-05	ns	FOCAD	LP	No	-	Involved in Colon rectal cancer, glioma
chr10	101775209	G	A	SNV	rs137852660	Het	0.41	8.4E-04	ms	FGF8	LP	No	CR	RAS related protein, CIS at RTCGD
chr6	32041006	C	T	SNV	rs6445	Het	0.31	5.1E-03	ms	CYP21A2/TNXB	LP	No	-	Matrix protein
chr16	23090623	C	T	SNV	rs770939342	Het	0.45	4.0E-06	sp	USP31	LP	No	-	Ubiquitin protease, CIS at RTCGD
chr2	47799169	C	G	SNV	rs2020908	Het	0.39	5.4E-03	ms	FBXO11/MSH6	B	Yes	CR	Repair protein, T-ALL and testis carcinoma
chr1	26779842	G	A	SNV	rs201604768	Het	0.55	3.4E-04	ms	ARID1A	B	Yes	CR	Transcription factor, tumor suppressor, AML
chr7	2924339	C	T	SNV	rs755710637	Het	0.40	8.0E-06	ms	CARD11	US	Yes	CR	TCR signal, CLL
chr11	119285447	C	A	SNV	rs763666786	Het	0.41	4.0E-06	ms	CBL	LB	Yes	CR	AML, Involved in CAR-T cell expansion
chr20	37397806	C	T	SNV	rs148049198	Het	0.53	1.0E-04	ms	SRC	US,LP	Yes/Low	CR	RTK pathway protein, colon cancer and AML
chr18	45644096	C	A	SNV	rs992886597	Het	0.31	4.0E-06	ns	SLC14A2	US, P	No	-	Urea transporter
chr19	39100964	C	T	SNV	rs767440775	Het	0.38	1.6E-05	ms	ACP7	US, P	No	-	Myxopapillary Ependymoma disease gene
chr9	127726291	C	T	SNV	rs770745688	Het	0.42	8.0E-06	ns	TTC16	US, P	Yes/Low	-	Uncharacterized protein, CIS at RTCGD
chr7	5228211	G	A	SNV	rs547593467	Het	0.49	3.6E-05	ms	WIPI2	US, P	Yes	-	Component of the autophagy machinery
chr6	26468398	C	G	SNV	rs201238249	Het	0.52	3.7E-04	ns	BTN2A1	US, P	Yes	-	Involved in lipid, fatty-acid, and sterol metabolism
chr1	26029665	G	A	SNV	rs141225406	Het	0.39	1.0E-03	ns	EXTL1	US, P	Yes/Low	-	Polymerization of heparan sulfate, breast, neuroblastoma
chr5	74751403	C	G	SNV	rs1743700822	Het	0.51	unknown	ms	GFM2	US,LP	Yes	-	Mitochondrial GTPase, solid cancer
chr8	85480739	C	A	SNV	rs745556921	Het	0.48	1.6E-05	ms	CA2	US,LP	Yes/Low	-	lsozymes catalyzes carbon dioxide reaction, invasion, Osteopetrosis
chr2	107859172	G	A	SNV	rs1306583979	Het	0.44	3.7E-03	ns	RGPD4	US, P	No	-	GTPase activator activity

Chr: Chromosome; Mutation type: SNV: single nucleotide variation; Del: deletion; Mutation impact: ns: nonsense; sp: altered splicing; ms: missense; Frequency: allele frequencies of each mutation's presence in the human population (gnomAD_exome); Predicted variant function: P: pathogenic, disease-causing; LP: likely pathogenic; US: unknown significance; B: benign: LB: likely benign; Het: Mutation in Heterozygosity; Gene role: CR: cancer-related gene; CLL: Chronic Lymphocytic Leukemia, AML: Acute Myeloid Leukemia; CIS at RTCGD: Identified as Common Insertion Sites in the Retroviral Tagged Cancer Gene Databases; RNAseq: expressed in blast cells as observed from RNAseq data

Supplementary Table 6. Main variables from γRV GT clinical trials for different IEI that may impact the tumorigenic outcome

First author	Year	Country	Clinical trial code/reference	N° Patients*	Disease	Vector LTR	GC within LTR sequence	Other vector features	Conditioning	OS (%)	Leuk (%)	Deaths	Follow-up months	CD34+ cells 10^6/kg median (min-max)	VCN
													Median (min-max)		min-max
Candotti[1]	2012	USA	NCT00018018	10	ADA-SCID	MPSV/ MND	54%	-	No/non-myelo	100	0	0	60 (30-120)	1.9 (0.7-9.8)	0.1-1.46
Reinhardt[2]	2021	USA	NCT00794508	10	ADA-SCID	MND	54%	-	non-myelo	100	0	0	108 (96-132)	6.23 (0.6-8.4)	0.6-2.68
Migliavacca[3]	2023	Italy	NCT00598481 NCT03478670	43	ADA-SCID	MoMLV	52%	SV40 prom/Neo	non-myelo	100	2.3	0	82.08 (2.92-266.97)	10.1 (0.9-25.7)	0.3-2.5
Gaspar[4]	2006	UK	-	1	ADA-SCID	SFFV	53%	mWPRE	non-myelo	100	0	0	24	1.4	-
Otsu[5]	2015	Japan	-	2	ADA-SCID	MND	54%	-	No	100	0	0	96 (72-120)	1.15 (0.92-1.38)	-
Kohn[6,7]	2008	USA	-	3	ADA-SCID	MoMuLV	53%	SV40 prom/Neo	No	100	0	0	180		-
Gaspar[8]	2011	UK	NCT01279720	6	ADA-SCID	SFFV	53%	mWPRE	non-myelo	100	0	0	43 (24-84)	1.65 (0.5-5.8)	-
Six[9-11]	2020	France, Australia	-	10	X-SCID	MoMuLV	53%	IL2RG	No	80	50	2	51-107	5 (1-22)	-
Gaspar[12]	2011	UK	-	10	X-SCID	MFG	53%	IL2RG	No	100	10	0	8-180	23.1 (6.9-34.1)	-
Chinen[13]	2007	USA	NCT00028236	3	X-SCID	MFG	53%	IL2RG	No	100	0	0	23 (12-30)	2.92 (2.85-3.13)	1.1-3.7
Thrasher[14]	2005	France, USA, UK	-	2	X-SCID	MFG	53%	IL2RG	No	100	0	0	6	18.9 (2.8-3.5)	-
Botzug[15,16]	2010	Germany	DRKS0000330	10	WAS	MoMLV	55%	WASP	non-myelo	70	90	3	15-86	18.25 (2.9-24.9)	1.7-3.2

MoMLV: Moloney murine leukemia virus; MPSV, myeloproliferative sarcoma virus; MND, modified myeloproliferative sarcoma virus enhancer (MND is ncr-deleted, coupled to dl587rev pbs; MPSV, MoMLV PBS); Leuk: Leukemia cases; non-myelo: non-myeloablative; MFG, a hybrid vector containing moloney murine leukemia virus (MoMLV) and myeloproliferative sarcoma virus (MPSV) LTR elements; Neo: Neomycin resistance; mWPRE: mutated version of the posttranscriptional regulatory element of woodchuck hepatitis virus (WPRE); MFG:; SFFV: Spleen Focus Forming Virus
*Since some articles may report the same patient the total number of patients may be different,

Transgene role and regulation:
ADA: purine metabolism, housekeeping, ubiquitous,
IL2RG: signal transduction, regulated, restricted expression (T/NK)
WASp: cytoskeleton reorganization and signal transduction, regulated, hematopoietic expression

Summary of main variables and outcomes from our study and publicly available information from other γ-RV GT clinical trials on ADA-SCID and other IEI. These include the disease background, the transgene, the cell (CD34+ cells/Kg) and vector dose (i.e. VCN in the drug product), the choice of enhancer/promoter

sequences in the vector (including GC content), the type of conditioning regimen. In pre-clinical models, lack of conditioning prior to transplantation or GT has been associated with an increased risk of leukemia, since the limited reconstitution of progenitors in the BM favored the proliferation and selection of a small number of transduced cell clones. Interestingly, most ADA-SCID patients treated with GT received busulfan at low intensity (4 mg/Kg), while SCID-X1 patients did not, pointing to a protective role for conditioning in the context of SCID GT, similarly to what has been observed in preclinical models. However, WAS patients, who developed T-ALL at high frequency, received busulfan conditioning at higher but still non-myeloablative doses, and no difference in actual exposure to busulfan has been observed between P21 and the other patients, thus indicating that chemotherapy is not a protective factor. Comparing the VCN in the drug product among the different trial we observed an higher VCN in the drug product (VCN 1.7-5.2) in WAS trial, which was reflected in a high VCN also in mature T cells *in vivo* due to the selective advantage conferred by gene correction, however VCN was similar in SCID-X1 and ADA-SCID. Moreover, no difference was found between VCN in P21 and the range observed in the other ADA-SCID patients from our cohort. Thus, VCN in the product is unlikely to have contributed. Similarly, no significant differences were detected in the in GC content within the LTR sequences of the γ–RV adopted in the different trials.

References

1 Candotti, F. *et al.* Gene therapy for adenosine deaminase-deficient severe combined immune deficiency: clinical comparison of retroviral vectors and treatment plans. *Blood* **120**, 3635-3646 (2012). https://doi.org:10.1182/blood-2012-02-400937

2 Reinhardt, B. *et al.* Long-term outcomes after gene therapy for adenosine deaminase severe combined immune deficiency. *Blood* **138**, 1304-1316 (2021). https://doi.org:10.1182/blood.2020010260

3 Migliavacca, M. Abstract #7. Gene Therapy for Adenosine Deaminase Deficiency: Long-Term Outcome and Post-Marketing Experience. *Mol Ther* **31**, 1-794 (2023).

4 Gaspar, H. B. *et al.* Successful reconstitution of immunity in ADA-SCID by stem cell gene therapy following cessation of PEG-ADA and use of mild preconditioning. *Mol Ther* **14**, 505-513 (2006). https://doi.org:10.1016/j.ymthe.2006.06.007

5 Otsu, M. *et al.* Outcomes in two Japanese adenosine deaminase-deficiency patients treated by stem cell gene therapy with no cytoreductive conditioning. *J Clin Immunol* **35**, 384-398 (2015). https://doi.org:10.1007/s10875-015-0157-1

6 Kohn, D. B. *et al.* T lymphocytes with a normal ADA gene accumulate after transplantation of transduced autologous umbilical cord blood CD34+ cells in ADA-deficient SCID neonates. *Nat Med* **4**, 775-780 (1998). https://doi.org:10.1038/nm0798-775

7 Schmidt, M. *et al.* Clonality analysis after retroviral-mediated gene transfer to CD34+ cells from the cord blood of ADA-deficient SCID neonates. *Nat Med* **9**, 463-468 (2003). https://doi.org:10.1038/nm844

8 Gaspar, H. B. *et al.* Hematopoietic stem cell gene therapy for adenosine deaminase-deficient severe combined immunodeficiency leads to long-term immunological recovery and metabolic correction. *Sci Transl Med* **3**, 97ra80 (2011). https://doi.org:10.1126/scitranslmed.3002716

9 Six, E. *et al.* Clonal tracking in gene therapy patients reveals a diversity of human hematopoietic differentiation programs. *Blood* **135**, 1219-1231 (2020). https://doi.org:10.1182/blood.2019002350

10 Ginn, S. L. *et al.* Treatment of an infant with X-linked severe combined immunodeficiency (SCID-X1) by gene therapy in Australia. *Med J Aust* **182**, 458-463 (2005).

11 Hacein-Bey-Abina, S. *et al.* Efficacy of gene therapy for X-linked severe combined immunodeficiency. *N Engl J Med* **363**, 355-364 (2010). https://doi.org:10.1056/NEJMoa1000164

12 Gaspar, H. B. *et al.* Long-term persistence of a polyclonal T cell repertoire after gene therapy for X-linked severe combined immunodeficiency. *Sci Transl Med* **3**, 97ra79 (2011). https://doi.org:10.1126/scitranslmed.3002715

13 Chinen, J. *et al.* Gene therapy improves immune function in preadolescents with X-linked severe combined immunodeficiency. *Blood* **110**, 67-73 (2007). https://doi.org:10.1182/blood-2006-11-058933

14 Thrasher, A. J. *et al.* Failure of SCID-X1 gene therapy in older patients. *Blood* **105**, 4255-4257 (2005). https://doi.org:10.1182/blood-2004-12-4837

15 Braun, C. J. *et al.* Gene therapy for Wiskott-Aldrich syndrome--long-term efficacy and genotoxicity. *Sci Transl Med* **6**, 227ra233 (2014). https://doi.org:10.1126/scitranslmed.3007280

16 Boztug, K. *et al.* Stem-cell gene therapy for the Wiskott-Aldrich syndrome. *N Engl J Med* **363**, 1918-1927 (2010). https://doi.org:10.1056/NEJMoa1003548
