# Supplementary material for: A case of T-cell acute lymphoblastic leukemia in retroviral gene therapy for ADA-SCID
Source: Nat Commun. 2024 Apr 30;15:3662. doi: 10.1038/s41467-024-47866-5 (PMC11061298; doi:10.1038/s41467-024-47866-5)
Supplement: Supplementary file 3 — Description of Additional Supplementary Files [file 41467_2024_47866_MOESM3_ESM.docx]

**Description of Additional Supplementary Files**

Supplementary Data 1_Single cell analyses

**Description:** The table includes two types of information: the cluster composition analysis with the frequency of cells belonging to each cluster within each condition (Cluster % frequency within condition sheet) and the results obtained from FindAllMarkers function used to identify markers for each cluster (from 0 to 30). The name of each sheet encompasses the number of the cluster and the classification label according to markers inspection. The columns of each sheet represent for each gene (column G): the p-value of Wilcoxon Rank Sum test and the adjusted p-value according to Benjamini-Hochberg (BH) correction for multiple tests (columns A and E), the average log fold change between expression values in the cluster (column F) vs all others, the percentage of cells expressing the gene in the cluster and in all the other clusters (columns C and D respectively).
